# Mental Health, Culture and Resilience—Approaching the COVID-19 Pandemic From a South African Perspective

**DOI:** 10.3389/fpsyt.2021.611108

**Published:** 2021-07-07

**Authors:** Sibongile Mashaphu, Mvuyiso Talatala, Sebolelo Seape, Lennart Eriksson, Bonginkosi Chiliza

**Affiliations:** ^1^Department of Psychiatry, University of KwaZulu-Natal, Durban, South Africa; ^2^Department of Psychiatry, University of the Witwatersrand, Johannesburg, South Africa

**Keywords:** COVID 19, spiritualty, resilience, religion, culture

## Abstract

It is understandable that the challenges of living through a severe contagious outbreak, like the coronavirus disease 2019 (COVID-19), cannot be tolerated for long and that some individuals may require emotional, psychological, and spiritual support in order to strengthen their resilience to navigate this difficult period. As clinicians and researchers in the field of mental health, we need to appreciate the roles that culture, spirituality, and religion play in comforting people who survive such an outbreak and provide possible solutions for public health authorities on how to promote wellness. This appreciation should direct us to seek a deeper understanding of how culture, spirituality, and religion can be used to endure an outbreak of this magnitude and how the interruption of common practices can impact the coping skills of those who are affected. Our understanding of the roles that customs, beliefs, and values of South Africans play in building resilience will help inform and strengthen interventions that are aimed at controlling the spread of COVID-19.

## Introduction

The coronavirus disease 2019 (COVID-19) is a high-impact widespread pandemic, with an imperfectly understood mode of transmission, poorly elucidated course, and a case fatality that has been ~2% in South Africa (SA) and an estimated fatality rate of 5–20% worldwide, with country-specific rates varying from 0.5 to 3.6% (1–3). Frontline health workers and policy makers have been left perplexed by this disease with regard to its evolution over time and the treatment approaches. We, irrespective of age, gender, or background, are all affected by this so-called invisible enemy. The COVID-19 pandemic, in addition to the mortality and physical morbidity, poses threats to the mental health of the entire population. The disease was first identified in a wholesale market in Wuhan, China, in December 2019 and runs a particularly aggressive course in those with underlying comorbidities such as obesity, diabetes, hypertension, cardiac disease, renal disease, and cancer (4, 5). To this day, several millions of people worldwide have been infected, while a few millions have succumbed to this virus. COVID-19, compared to other epidemics such as HIV, severe acute respiratory syndrome (SARS), Middle East respiratory syndrome (MERS), Ebola, and H1N1, has run a more severe course because of a rapid spread that resulted in acute morbidity and mortality.

With the current reports of new mutations that may affect vaccine efficacy in several countries (6) and predictions of possible surges or waves in certain clusters, it is clear that this virus is likely to stay with us for some time. Therefore, non-pharmacological measures remain the most reliable long-term strategies to control the pandemic (7). In order to reduce the rate of transmission, the strictest measures of public health prevention and infection control are being applied. These measures include strict hygiene routines, self-isolation, quarantine, movement restriction, and social distancing, which were introduced on various communities by most governments with little consideration to the mental, social, physical, and economic preparedness of individuals and families (8). Several countries, including the UK and USA, have established procedures for psychological crisis interventions to deal with public health emergencies (9). Similarly, our country needs to develop similar guidelines that can be used to strengthen mental health initiatives during a time of crisis without fuelling the spread of the virus. Such protocols should be relevant to the local context because sociocultural practices are the distinctive spiritual material that characterizes a society as a social group (10).

In this paper, we explore the COVID-19 pandemic from a South African perspective by highlighting the impact of this pandemic on certain social norms and linking the important role of culture, spirituality, and religion in promoting mental health and resilience. Furthermore, we provide perspectives on how individuals and societies can be supported to continue to practice their spiritual and religious activities without breaking the prescribed guidelines.

## The Pandemic Poses Challenges to Common Spiritual and Religious Practices

The management of a patient with COVID-19 drastically deviates from how people have been accustomed to looking after those who are suffering from a medical illness. In SA, the regulations of the Disaster Management Act No. 57 of 2020 prohibit the visitation of the sick in hospital, thereby preventing any physical contact the families may have with those with COVID-19 (11). Families of hospitalized patients with COVID-19 and other illnesses do not get an opportunity to communicate with their loved ones even in times of imminent death, missing opportunities to practice rituals of dealing with those who are sick and dying that have been established over centuries. Even those who do not receive care in health facilities are subjected to strict quarantine and self-isolation for long periods of time (12).

COVID-19 deaths have been perceived by many as cold and inhumane in nature, forcing frontline workers to make quick decisions about end-of-life care while shielding vulnerable family members from getting the disease (13). The respondents of a UK-based study that was conducted on individuals who were bereaved during the pandemic highlighted the benefit of after-death rituals, including funeral attendance, as a meaningful way for the bereaved to pay their last respects (14). Visiting the sick, offering a prayer, and performing rituals for the sick and those who are dying are practices common to most societies, and abandoning these may result in complex bereavement issues, as described by some countries that experienced the pandemic before us (15–17). In the South African context, the practice of animistic and Christian rituals that have been passed down from generation to generation is believed to have a protective and emotionally uplifting role for the bereaved (18). The lockdown restrictions prohibit large gatherings, which means that some close friends and family members will not have the opportunity to mourn the loss of a loved one according to usual practices (19).

These restrictions forced our communities to find new ways to connect without violating the set restrictions. The use of virtual or online platforms turned out to be a plausible solution to this problem; however, in low and middle-income countries, resource limitations mean that many people do not have access to smartphones, Internet connectivity, or data. The WHO published guidelines on avoiding large faith group gatherings and encouraged conducting rituals and faith-related activities remotely or virtually (20), but the practical application of these guidelines remains to be established through research. In other countries such as the UK and Ireland, the non-contact ways of dealing with bereavement were assessed, and caution has been made to avoid the “tsunami of grief” by promoting support services for the bereaved (17). Events that are time-sensitive and cannot be postponed until the pandemic is under control, such as funerals, were performed under the new norms, but other cultural practices that are not time-sensitive may be postponed to a later time; for example, the winter schools for the rites of passage of initiation of boys to manhood were postponed to the summer season due to COVID-19^1^. Illegal practitioners of initiation schools have already taken advantage of this postponement and ran illegal schools. In the long term, there could be a psychological impact in boys who grow older and feel delayed in their passage to manhood^2^.

## Public Health Information

The way in which most people have embraced the new social distancing norms without fully understanding the pathogenesis of this virus or experiencing it first hand is admirable. As of now, there is no definitive antiviral for this disease, but several vaccine candidates are being rolled out worldwide (21, 22). This implies that COVID-19 is one of those dreaded medical conditions that do not yet have a cure, but can be prevented by a combination of mass vaccination programs and non-pharmacological measures such as hand hygiene and social distancing. This may pose some serious challenges to those who strongly believe in divine interventions, fueling debates on science vs. spirituality in health-related matters, so to speak. In managing a pandemic, it is important to contextualize this point so that public health information is packaged in a way that is clear, accurate, and culturally sensitive in order to provide specific health promotion and disease prevention, more so when health guidelines strongly contradict normal cultural practices. For example, funerals and church gatherings that resulted in the spread of the infection during the first wave in the South African arm of the pandemic highlight the difficulties that communities have experienced in adapting to this “new normal” way of processing grief, but provided the public with some insights on how large gatherings fuel the spread of COVID 19^3^. Within the continent, the COVID-19 fight in Uganda demonstrated that religion and its institutions are instrumental in mobilizing citizens to abide by government programs, especially public health programs (20).

Table 1 shows the contrast between common cultural practices and the COVID-19 regulations. As clinicians and researchers in the field of mental health, it is imperative that we change the narrative to reaffirm that staying away from a funeral and reducing the number of attendees are the best forms of support you can show during these times. These health messages need to be consistent and evidence-based in order to provide the most updated scientific information that will promote adherence to the new regulations (23).

**Table 1:** Contrast between common social practices and COVID-19 regulations.

**Cultural beliefs**	**COVID-19 regulations**
Group activities	Social distance
Seeking closeness during illness	Self-isolation
Shaking hands	No touching
Unity in numbers	Staying away is caring
Washing of mortal remains	No contact with mortal remains

## The Role of Spirituality and Religion In Promoting Mental Health During a Pandemic

Spirituality and religion are an important resource for well-being. Many researchers have demonstrated that spirituality plays a significant role in the lives of people, their thoughts, and behaviors (24–26). Spirituality provides a framework of meaning for people in their daily lives, as well as during major life crises. Similarly, religious practices form part of the organized and institutional components of faith traditions, which are common to our society (27). In SA, taking part in religious, spiritual, and cultural rituals is generally done in groups. This is a normal social practice through which individuals experience a sense of cohesiveness and belonging, and restricting these may translate into loss of routine pleasurable social activities. For example, identified places where people usually access social support, like churches, schools, and sporting venues, have limited access during hard lockdowns. In a multicultural and ethnically diverse country like SA, there is bound to be a resultant breakdown and significant loss of the social support systems that promote resilience, thus making people susceptible to psychosocial distress and mental health problems.

According to indigenous knowledge systems, African spirituality has emphasized that life is interconnected and that, in order to be fully human, one needs to address the holistic development of a person's physical, mental, social, economic, and spiritual well-being (28). Social connections have thus been identified as a key platform wherein individuals are protected against changes to mental status and behavior (26). The enforcement of social distancing has led to alterations in connections with friends and family members, factors shown to be protective against mental health disorders and to foster resilience in the face of adversity. We can draw lessons from the concurrent HIV epidemic, which is running a parallel course with COVID-19 in SA. During the devastating spread of HIV, religious, spiritual, and cultural beliefs played a significant role in mitigating against mental health problems among sufferers, their families, and healthcare workers (29).

## Suggestions for Overcoming the Current Limitations

According to media reports in SA, gatherings of large groups of people have been responsible for the majority of community spread, the so-called super spreader events^3^; e.g., the first outbreak in the Free State province occurred following a church gathering. The WHO has repeatedly cautioned that a pandemic like COVID-19 will not be defeated by only scaling up health facilities and resources but also through a change of behavior (20). Our communities have the responsibility to reduce the spread of this virus by following the non-pharmacological approaches that have been circulated on various public health platforms. Getting individuals to take responsibility for themselves for the good of everyone else is an important message to spread across our communities. The concept of *Ubuntu* (30), which is defined as humanness—a pervasive spirit of caring and community, harmony and hospitality, respect and responsiveness—that individuals and groups display for one another is strongly encouraged. An organizing concept of Ubuntu is human interdependence, and the driving norms are reciprocity, suppression of self-interest, and the virtue of symbiosis. These concepts can be used in specific conversations about the experiences of being exposed to a pandemic outbreak, with the aim of reinforcing the relational interactions between self-protection and community protection within a cultural context, like it was done in HIV prevention programs (31).

The issue of society, culture, and religious practices in a changing world deserves special mention within the African context, which is characterized by inequities and vulnerabilities. In adapting to the new norms, we should promote and use innovation to enhance social connection and ritual effectiveness, as was the case in Uganda (32). Many interesting developments have already transformed how rituals are performed, e.g., funerals are livestreamed, highlighting the value of using technology for establishing connection during religious services and funerals. Sadly, in most African communities, access to technology and other resources that are required to promote these new initiatives is not available due to resource constraints as well as social and racial inequalities. The recommendations published by the WHO encourage the use of virtual platforms, but how these ideas should be operationalized in resource-constrained settings remains to be established through research (20).

We have borrowed from the West African culture the principles of Nguzo-Saba (unity, self-determination, collective work and responsibility, cooperative economics, purpose, creativity, and faith), which have been repeatedly used in public health interventions such as HIV to motivate behavior changes in order to prevent the spread of the virus within communities (33). Using the same principles, aligned with the basic concept of Ubuntu, we propose a model to utilize as a public health tool that clearly defines the culturally relevant role of each individual toward reducing the spread of COVID-19. Table 2 below shows the application of the concept of Ubuntu in reducing the spread of COVID-19, which can be used to shape public health messages. It is anticipated that when the large-scale rollout of vaccines occurs, further, challenges will be experienced in terms of the distribution to our large population. Health authorities have published clear guidelines of the vaccine rollout plans, starting with our most vulnerable populations and frontline workers, but in South Africa, there have been anecdotal reports of vaccine fraud among healthy individuals who had the vaccine before their turn (34). This process will require the application of some of the Ubuntu concepts and principles, where individuals with no vulnerabilities will receive the vaccine at a later stage, and vaccine hesitancy can be discouraged in the same manner. Moreover, the rollout of the vaccine depends on technology, with registrations taking place online. It is through the application of the same Ubuntu principles that we can encourage individuals to support those who are unable to use these technologies or have no access to such so that they are not left out.

**Table 2:** Applying the concept of Ubuntu in response to a pandemic.

**Concept of Ubuntu and the Nguzo-Saba principles**	**Application in response to a pandemic**
Unity	Encourage others to adhere to social distancing. Support others from a distance. Do not stigmatize those with COVID 19 symptoms.
Self-determination	We all have a role to play in stopping the pandemic. Wear a mask when in public. Adhere to social distancing. Protect the vulnerable individuals. Disclose your symptoms.
Collective work and responsibility	Share essential resources, such as water, face masks, and hand sanitizers. Protect your family. Protect your colleagues. Protect your community. Save lives. Take the vaccine.
Cooperative economics	Sharing of resources. Allocating more resources to vulnerable individuals.
Purpose	To work together toward a common goal to end the pandemic.
Creativity	Use innovative ways to connect. Use technology to provide essential services.
Faith	The virus shall be defeated if we all play our part.

## Highlighting Important Research Issues

A large number of questions remain unanswered because the virus has only been around for a short period of time and the long-term effects of social isolation are yet to be experienced and reported on. Going forward, it will be interesting to look into the sustainability of the new norm and to observe whether people will return to the old ways of doing things when the pandemic is over. The interconnections of life appear more overtly during cultural rituals, such as birth rites, coming-of-age ceremonies, weddings, and funerals, than at any other times, and any noticeable changes in these practices should be duly documented because animistic rituals are intergenerational and may become fluid and change with time (35). At times like these, when change is forced upon the entire population, opportunities may arise for us to explore our indigenous knowledge systems for fluidity, flexibility, and resilience.

## Conclusion

This pandemic has no end in sight yet, and according to the WHO, the earliest we are likely to go back to normal practices will be in the middle of the year 2022. In the meantime, we need to adapt and thrive under the new normal living conditions by promoting social, spiritual, and cultural activities while conforming to the stipulated COVID-19 guidelines.

## Data Availability Statement

The original contributions generated for the study are included in the article/supplementary material, further inquiries can be directed to the corresponding author/s.

## Author Contributions

All authors listed have made a substantial, direct and intellectual contribution to the work, and approved it for publication.

## Conflict of Interest

The authors declare that the research was conducted in the absence of any commercial or financial relationships that could be construed as a potential conflict of interest.

## Footnotes

^1^www.sanews.gov.za.

^2^https://www.groundup.org.za/article/covid-19-circumcision-schools-suspended-eastern-cape/.

^3^www.healthline.com>health-news.
